# The rearrangement of actin cytoskeleton in mossy fiber synapses in a model of experimental febrile seizures

**DOI:** 10.3389/fneur.2023.1107538

**Published:** 2023-04-20

**Authors:** Nuo Yang, Yin-Bo Chen, Yan-Feng Zhang

**Affiliations:** ^1^Department of Pediatric Neurology, The First Hospital of Jilin University, Changchun, China; ^2^Jilin Provincial Key Laboratory of Pediatric Neurology, Changchun, China

**Keywords:** febrile seizures, filamentous actin, synaptic remodeling, hippocampus, hippocampal hyperexcitability

## Abstract

**Background:**

Experimental complex febrile seizures induce a persistent hippocampal hyperexcitability and an enhanced seizure susceptibility in adulthood. The rearrangement of filamentous actin (F-actin) enhances the excitability of hippocampus and contributes to epileptogenesis in epileptic models. However, the remodeling of F-actin after prolonged febrile seizures is to be determined.

**Methods:**

Prolonged experimental febrile seizures were induced by hyperthermia on P10 and P14 rat pups. Changes of actin cytoskeleton in hippocampal subregions were examined at P60 and the neuronal cells and pre- /postsynaptic components were labeled.

**Results:**

F-actin was increased significantly in the stratum lucidum of CA3 region in both HT + 10D and HT + 14D groups and further comparison between the two groups showed no significant difference. The abundance of ZNT3, the presynaptic marker of mossy fiber (MF)-CA3 synapses, increased significantly whereas the postsynaptic marker PSD95 did not change significantly. Overlapping area of F-actin and ZNT3 showed a significant increase in both HT+ groups. The results of cell counts showed no significant increase or decrease in the number of neurons in each area of hippocampus.

**Conclusion:**

F-actin was significantly up-regulated in the stratum lucidum of CA3, corresponding to the increase of the presynaptic marker of MF-CA3 synapses after prolonged febrile seizures, which may enhance the excitatory output from the dentate gyrus to CA3 and contribute to the hippocampal hyperexcitability.

## 1. Introduction

Febrile seizures (FSs) are the most common convulsions during childhood, affecting 3–5% of infants and young children (1). In more than one third of these children the FSs are prolonged or recurrent, that is, complex FS, which raises the risk of mesial temporal lobe epilepsy (MTLE) in later life (2–4).

Experimental complex FSs induce a persistent hippocampal hyperexcitability, which leads to an enhanced susceptibility to seizures in adulthood (5). That is, the model of complex FSs does not have the spontaneous behavior or electrophysiological seizures during adulthood, but have a decreased seizure threshold to chemical convulsants or electrical stimulation, which is similar to the chemical kindling model of epilepsy (6).

The cellular mechanisms underlying the persistent hippocampal hyperexcitability after FSs in early life remain a focus to be intensely elucidated. The excitability of hippocampus is regulated by hippocampal trisynaptic circuit and among them MF-CA3 synapses play a decisive role due to the unique organization of pre- and postsynaptic structures (7–9). The remodeling of synapses has been demonstrated to be driven by the dynamics of actin, which is the main cytoskeleton component in both pre- and postsynapses (10, 11). The dynamic balance of actin between filamentous and monomeric (G-actin) states is delicately regulated by a series of events in the process of neuronal information transmission (11). Disturbance of this dynamic balance often leads to a pathological condition. Some studies reported the relationship between remodeling of F-actin cytoskeleton and epileptogenesis (12–16). Depolymerizing F-actin using latrunculin A and stabilizing F-actin using jasplakinolide in rat hippocampus both led to epileptic seizures and long-term neuronal hyperexcitability (12). Our previous studies also showed the changes of actin network in hippocampal MF-CA3 synapses in both PTZ kindling model and pilocarpine post status epilepticus model (13, 14). In addition, administration of FK506 or dexamethasone attenuated the damage of the actin cytoskeleton and synaptic structures and contributed to the reduction of spontaneous seizures in chronic period (15, 16). These indicate that the disturbance of actin network played a role in development of chronic epilepsy state and treatment targeting actin network may help to prevent epileptogenesis.

Therefore, we reasoned that examining the hippocampal synaptic actin network might provide some clues to the molecular mechanism for the hippocampal hyperexcitability after FSs. Some studies also clarified that the prognosis of febrile seizures occurring in younger children (less than 12 or 18 months) may be different and have a higher risk of recurrence and developing epilepsy (17–19). In this study, prolonged experimental FSs were induced by hyperthermia on P10 and P14 rat pups which corresponds human beings within and after 1-year-old, respectively, (20). Changes of F-actin cytoskeleton in subfields of hippocampus were examined and the neuronal cells and pre/postsynaptic components were labeled to determine changes of neuronal function after prolonged experimental FSs.

## 2. Materials and methods

### 2.1. Animals

The protocol of animal experiments was approved by the Research Ethics Committee of the First Hospital of Jilin University, Changchun, China (2020–0496). The principle of “Three Rs” was considered to design the experiments. All experiments of the present study were performed on male Wistar rats (ChangSheng Biotechnology Co., Ltd. China). In total, 100 male Wistar rats were used (20 for control, 40 for HT + 10D and 40 for HT + 14D). Five to 10 pups were housed together with the dam. After P21, the dam was removed from the nest. After P30, animals were housed individually. Housing rooms were quiet and temperature was controlled at 22–26°C, with a cycle of 12 h light and 12 h dark. Animals were allowed free access to food and water.

### 2.2. Prolonged experimental febrile seizures and group assignment

Prolonged experimental FSs were induced by hyperthermia as described earlier (21–23). To be brief, after a dose of 0.9% NaCl 0.2 ml subcutaneous injection to prevent dehydration, P10 and P14 pups were placed in a Perspex cylinder of 50 cm high, 10 cm diameter and subjected to a regulated stream of heated air. The Rectal temperature was measured every 2 min and a core temperature of 40–42°C was kept for 30 min. Behavioral seizures of animals were recorded by two observers. Seizures evoked by hyperthermia (HT+) characterized as limb clonus, rearing and falling, which belonged to stage 3–5 seizures based on Racine scale (24). The HT+ animals were further divided into HT + 10D and HT + 14D groups. Animals that did not display behavioral seizures during hyperthermia were classified as HT- group. After treatment of hyperthermia, animals were cooled down to the normal body temperature with a water-soaked tissue and returned to the dam. The control animals were subjected to the same treatment, except that they were kept normothermia of about 35°C (NT).

### 2.3. Sample preparations

Sample Preparations were performed as described earlier (13, 14). Briefly, at P60, rats were anesthetized with isoflurane, sacrificed, and perfused with 0.9% NaCl and 4% paraformaldehyde subsequently. Then the brains were dissected out and immersed in 4% paraformaldehyde overnight, followed by a series of sucrose solutions with increasing concentration (10, 20, and 30% sucrose in 0.1MPB) successively. Samples were embedded in the embedding agent (Tissue-Tek O.C.T. Compound), frozen on dry ice, and cut into 30-micron-thick serial slices coronally with a Leica cryostat (GmbH, Germany) for preparation of experiments.

### 2.4. F-actin labeling in hippocampal subfields

As described previously (13, 14), F-actin in subfields of hippocampus was specifically labeled by the phalloidin conjugated to Alexa 488 with free-floating staining. After incubation with Triton X-100 (pH 7.2, 0.3%) for 30 min, the sections were treated with Alexa Fluor™ 488 Phalloidin (A12379, Molecular Probes; 1:100) in the dark at 4°C overnight. Then sections were washed three times using 0.1 M PB and mounted with anti-fading reagent (Immu-Mount, Thermo Scientific, United States) for preparation of confocal scanning.

### 2.5. Neuron labeling in subfields of hippocampus

As described previously (16, 25), hippocampal neurons in subfields dentate gyrus (DG), CA3 and CA1 were labeled by anti-NeuN antibody. Slices were pretreated with 10% normal donkey serum containing 0.3% Triton X-100 at room temperature for 30 min and then were treated with monoclonal anti-NeuN antibody (ab177487, Abcam, United States; 1:100) at 4°C overnight. After that, slices were washed with 0.1 M PB three times, followed by incubation with donkey anti-rabbit secondary antibody labeled by Alexa 488 (A21206, Molecular Probes; 1:200) in the dark at room temperature for 2 h.

### 2.6. Labeling pre- and postsynaptic markers of mossy fiber synapses

To test the remodeling of mossy fiber synapses after prolonged febrile seizures， we labeled the pre- and postsynaptic components of mossy fiber synapses with ZNT3 and PSD95, respectively. Standard staining protocols for immunofluorescence with free-floating sections were used for ZNT3 and PSD95 labeling, which were mentioned in detail previously (16). The primary antibodies included rabbit anti-ZNT3 (No. 197002, Synaptic Systems; 1:200) and PSD-95/SAP90 (No. 51–6,900, Invitrogen, CA, United States; 1:100). For PSD95 detection, slices were pretreated with pepsin to increase PSD95 immunoreaction (26). For double staining of F-actin and ZNT3, phalloidin conjugated with Alexa 488 was co-incubated with Alexa 546 labeled secondary antibody (A20183, Molecular Probes; 1:200).

### 2.7. Image acquisition and data analysis

For confocal scanning and statistical analysis, 7–8 animals were randomly selected from HT + 10D, HT + 14D, and the control group, respectively. A Zeiss 710 confocal laser scanning microscope was used for fluorescence imaging experiments. The fixed confocal parameters were set for each experiment. Samples of each group including one control, one HT + 10D, and one HT + 14D were examined in the same day and the samples for each experiment were examined within several consecutive days as soon as possible.

A Plan-Apo 20 × objective (0.8 NA, WD 0.55 mm) was used to capture the region of CA1, CA3, and DG in the hippocampus. Then a Plan-Apo 63× oil immersion objective (1.4NA, WD 0.19 mm) was used to scan the interest area. The fluorescence signals of F-actin and ZNT3 double labeling were captured with two-channel confocal imaging simultaneously. Hippocampal slices were selected from anterior–posterior coordinates Bregma −3.3 to −4.8 mm. The selected slices in different groups were matched carefully.

**Figure 1:**
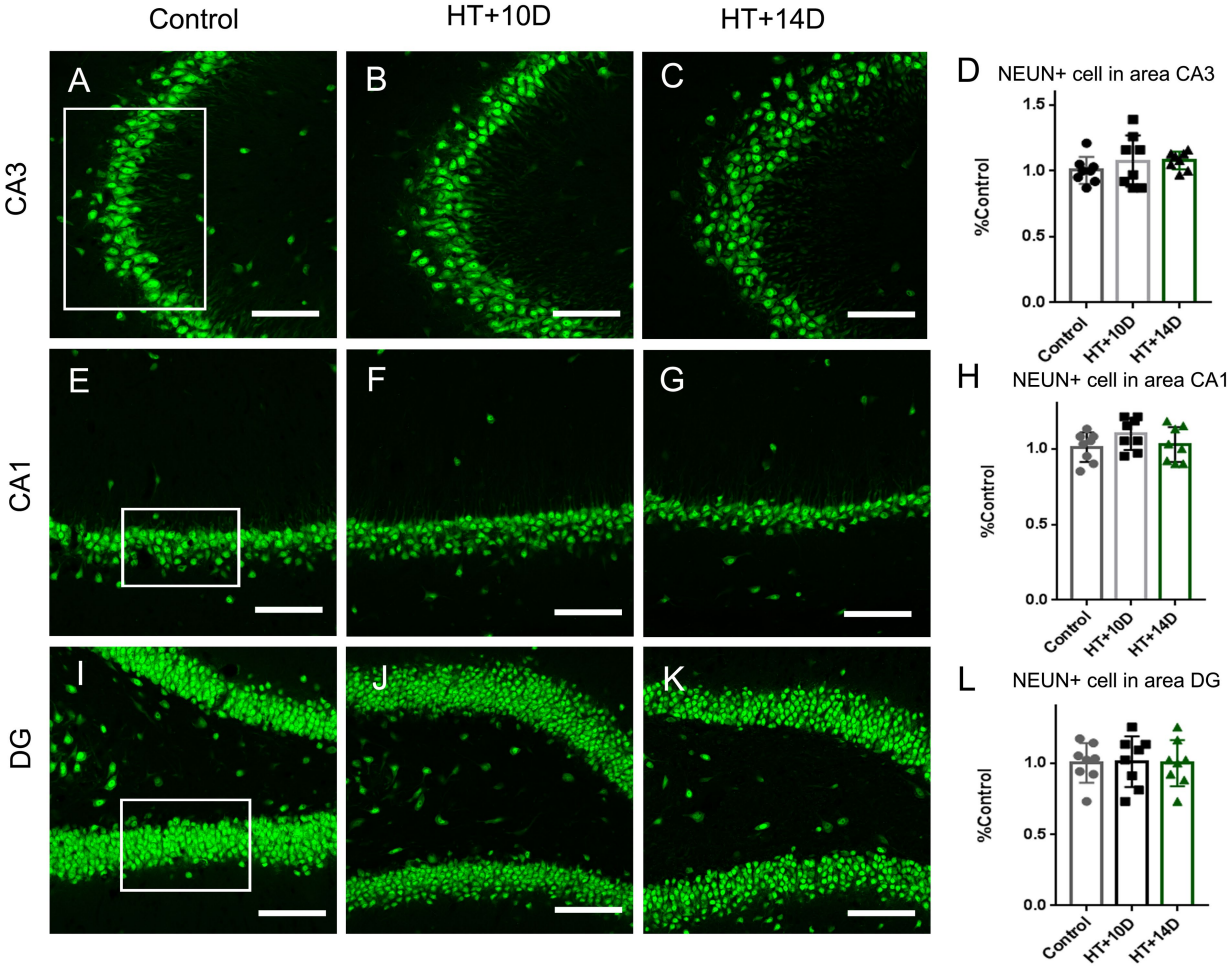
The number of NEUN positive neurons in subregions of hippocampus is unaltered after experimental febrile seizures. Scale bars: 100 μm. Compared with the control group, there is no remarkable change in the number of pyramidal neurons in subregion of CA3 **(A–D)** and CA1 **(E–H)**, as well as the granule cells in DG **(I–L)** after experimental febrile seizures in both HT + 10D and HT + 14D groups (*n* = 8 per group).

The labeling density of positive puncta of F-actin, ZNT3, PSD95 and overlapping area of F-actin and ZNT3 were examined by the software Image-Pro Plus analysis (v. 6.0, Media Cybernetics, Silver Spring, MD, United States). Images captured with the 63 × objective with a 2 × optical zoom were used. Four fields for each hippocampal subregion were measured in each sample and at least three sections were measured for each animal. Pyramidal cells in CA1 and CA3a and granule cells in DG were counted in a fixed field of rectangle shown in Figure 1 with Image J software and at least three sections were measured for each animal.

The normality of raw data was tested by SPSS25 software (IBM SPSS Statistics Version 25.0, United States). After that, the data were exported to GraphPad Prism 8 software (GraphPad Software, United States) for statistical analyses and figure preparation. Data with a normal distribution were presented as mean ± SD and the test of one-way ANOVA was performed, followed by *post hoc* Turkey test in multiple comparisons of three groups. Data with an abnormal distribution were presented as M (P25, P75). The nonparametric statistics of Kruskal–Wallis test was performed, followed by a Dann’s *post hoc* test for multiple comparisons. The significance set for statistical analysis was *p* < 0.05.

## 3. Results

### 3.1. F-actin cytoskeleton rearranging in hippocampal subfields after prolonged febrile seizures

**Figure 2:**
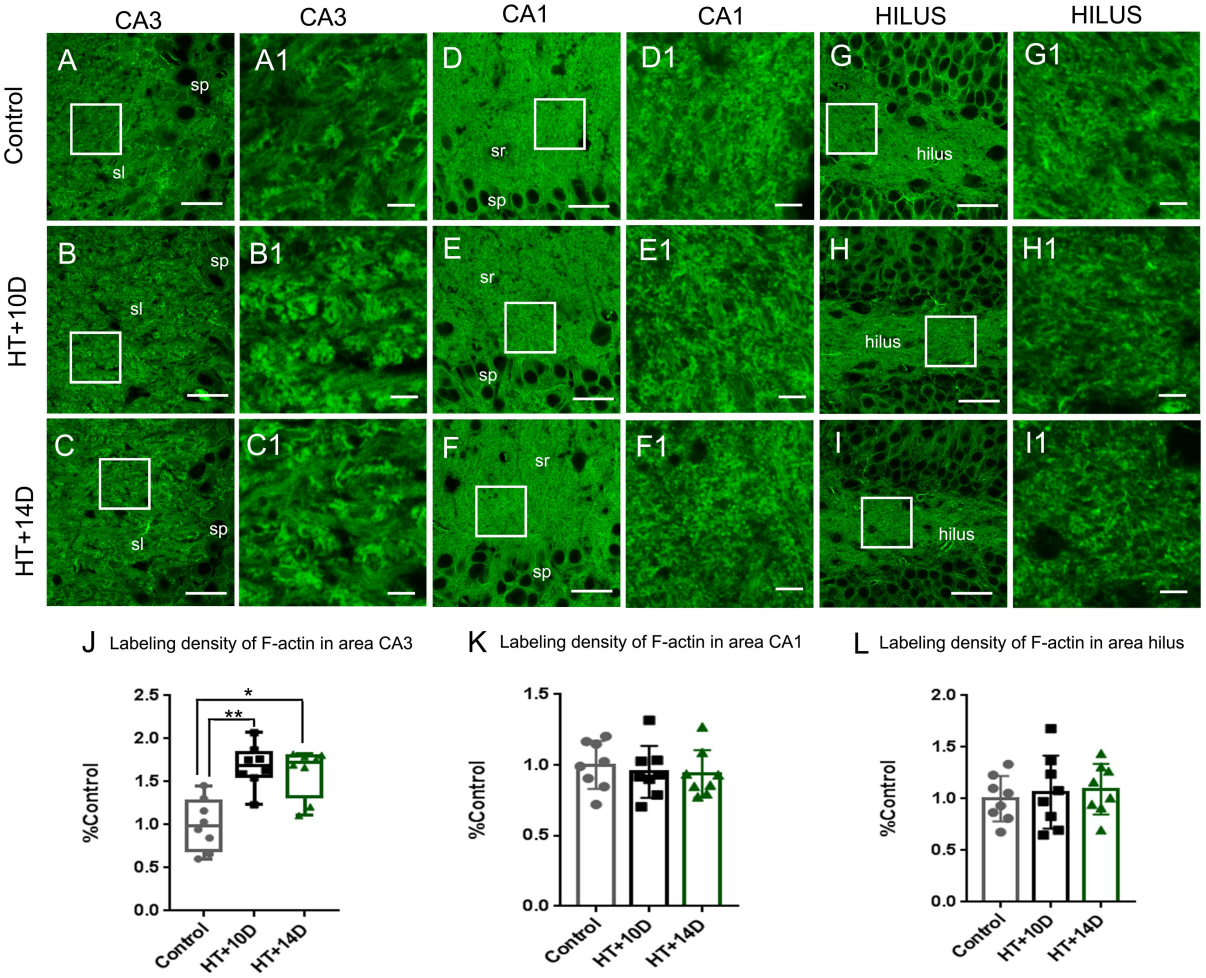
The rearrangement of F-actin cytoskeleton in subregions of hippocampus after experimental febrile seizures. Scale bars: **(A–I)** 30 μm; **(A1–I1)** 5 μm. Images **(A–I)** show the distribution of F-actin in the hippocampal CA3 region, CA1 region and hilus of DG in the control, HT + 10D and HT + 14D groups, respectively. Images **(A1–I1)** are enlarged from the insets of images **(A–I)**. Compared with that of control group **(A,A1)**, the labeling density of F-actin increases remarkably in the stratum lucidum of subregion CA3 after experimental febrile seizures (**B,B1** for HT + 10D, **C,C1** for HT + 14D, **J** for statistical analysis). While no significant difference is detected between the control group and HT+ groups in subregion CA1 (**E,E1** for HT + 10D, **F,F1** for HT + 14D, **K** for statistical analysis) and hilus of DG (**H,H1** for HT + 10D, **I,I1** for HT + 14D, **L** for statistical analysis). Further comparisons of F-actin labeling density in subfields CA3, CA1, and hilus of DG between group HT + 10D and HT + 14D show no significant difference (*n* = 8 per group; *p < 0.05, **p < 0.01).

To detect the rearrangement of F-actin cytoskeleton after prolonged febrile seizures, F-actin in subfields of hippocampus was labeled with phalloidin, which binds actin filamentous specifically but not actin monomers (G-actin). Compared with that of control group (Figures 2A, A1), the labeling density of F-actin increased remarkably in the stratum lucidum of subregion CA3 after experimental febrile seizures (Figures 2B, B1 for HT + 10D, Figures 2C, C1 for HT + 14D, Figure 2J for statistical analysis). The relative positive area of F-actin in the stratum lucidum of CA3 region in each group was as follows: Control 0.99 (0.17, 1.26), HT + 10D 1.69 (1.56, 1.84), HT + 14D 1.72 (1.32, 1.80) (**p1 = 0.0056, *p2 = 0.010, *n* = 8 per group). However, no significant difference is detected between the control group (Figures 2D, D1) and HT+ groups in subregion CA1 (Figures 2E, E1 for HT + 10D, Figures 2F, F1 for HT + 14D, Figure 2K for statistical analysis). The relative positive area of F-actin in the stratum radiatum of CA1 region in each group was as follows: Control 1.00 ± 0.17, HT + 10D 0.95 ± 0.18, HT + 14D 0.94 ± 0.17 (*p*1 = 0.84, *p*2 = 0.75, *n* = 8 per group). Similarly, no significant difference is detected between the control group (Figures 2G, G1) and HT+ groups in hilus of DG (Figures 2H, H1 for HT + 10D, Figures 2I, I1 for HT + 14D, Figure 2L for statistical analysis). The relative positive area of F-actin in the hilus of DG in each group was as follows: Control 1.00 ± 0.22, HT + 10D 1.06 ± 0.35, HT + 14D 1.09 ± 0.24 (*p*1 = 0.89, *p*2 = 0.78, *n* = 8 per group). Further comparisons of F-actin labeling density in subfields CA3, CA1, and hilus of DG between group HT + 10D and HT + 14D were performed and no significant difference was detected (*p*1 = 0.99, *p*2 = 0.99, *p*3 = 0.98. *n* = 8 per group).

### 3.2. The number of neurons in hippocampal subfields was unaltered after prolonged febrile seizures

To determine whether the rearrangement of F-actin cytoskeleton in subfields of hippocampus is due to the altered number of neurons, the immunofluorescence staining of anti-NeuN was conducted. Compared with the control group, there is no remarkable change in the number of pyramidal neurons in subregion of CA3 (Figures 1A–D) after experimental febrile seizures in both HT + 10D and HT + 14D groups. The relative cell count of NEUN+ pyramidal cells in CA3 region in each group was as follows: Control 1.00 ± 0.10, HT + 10D 1.08 ± 0.20, HT + 14D 1.08 ± 0.07 (*p*1 = 0.54, *p*2 = 0.51, *n* = 8 per group). Similarly, no significant difference is detected between the control group and HT+ groups in the number of pyramidal neurons in subregion of CA1 (Figures 1E–H). The relative cell count of NEUN+ pyramidal cells in CA1 region in each group was as follows: Control 1.00 ± 0.10, HT + 10D 1.10 ± 0.10, HT + 14D 1.03 ± 0.12 (*p*1 = 0.25, *p*2 = 0.94, *n* = 8 per group). Besides, no significant difference is detected between the control group and HT+ groups in the number of the granule cells in DG (Figures 1I–L). The relative cell count of granule cells in DG in each group was as follows: Control 1.00 ± 0.14, HT + 10D 1.01 ± 0.18, HT + 14D 1.00 ± 0.16 (*p*1 = 0.99, *p*2 = 0.99, *n* = 8 per group).

### 3.3. Remodeling of mossy fiber synapses after prolonged febrile seizures

Mossy fibers project primarily to the hippocampal subfield CA3. We then examined whether the pre/postsynaptic structures of mossy fiber synapses were affected after prolonged febrile seizures.

**Figure 3:**
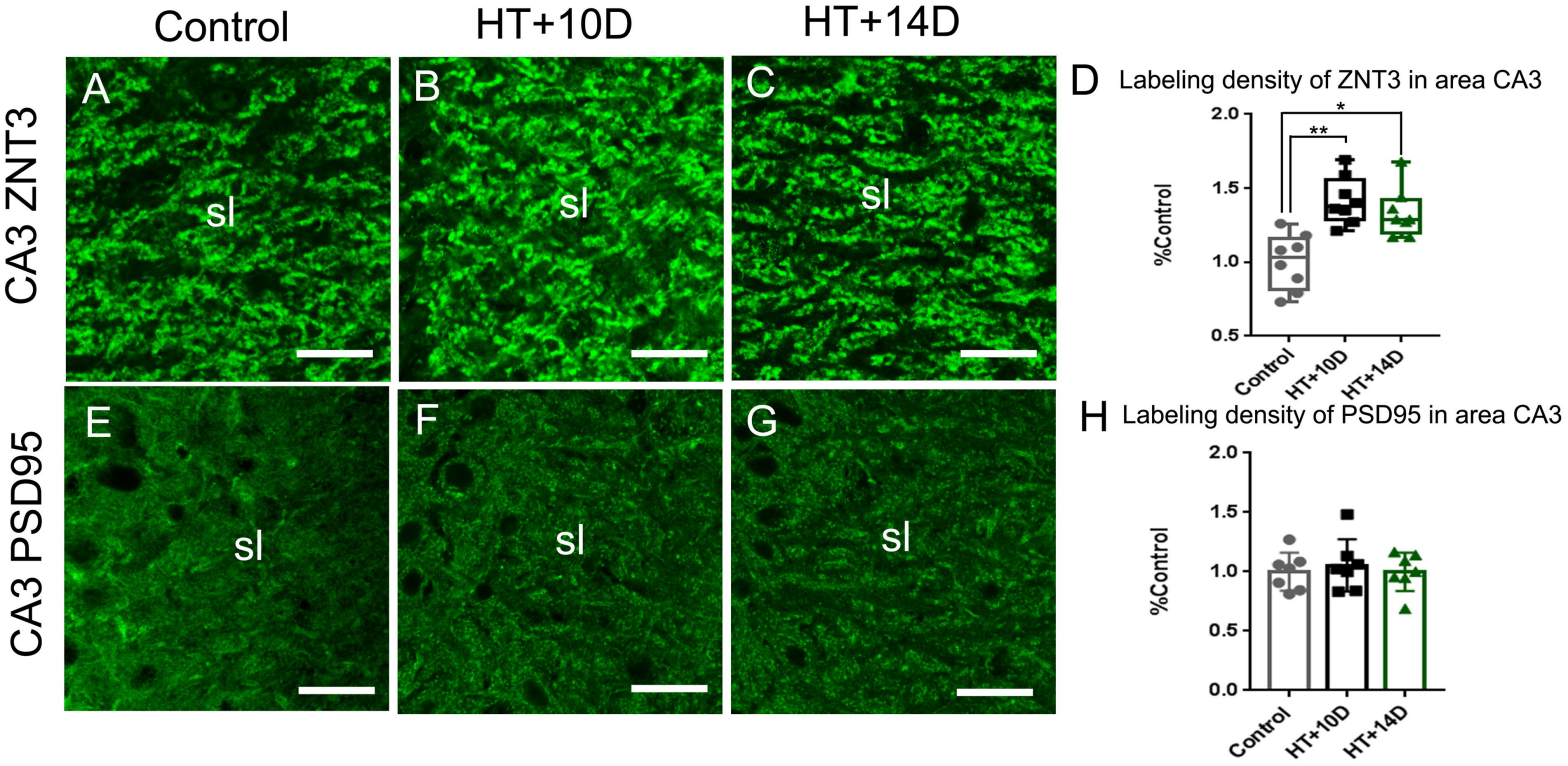
Remodeling of pre- and postsynaptic markers of MF-CA3 synapses after experimental febrile seizures. Scale bars: 25 μm. Compared with the control group, the labeling intensity of ZNT3, the mossy fiber presynaptic marker, increase significantly in subfields CA3 **(A–D)** in both HT + 10D and HT + 14D groups (*n* = 8 per group; **p < 0.01, *p < 0.05). While PSD95, the postsynaptic marker, is not remarkably altered in subfields CA3 **(E–H)** in both HT + 10D and HT + 14D groups (*n* = 7 per group).

The presynaptic component of mossy fiber synapses, namely large mossy fiber terminals (LMTs), belong to the most efficient synaptic structures in the brain. The LMTs are rich in zinc and ZNT3, the most important zinc transporter, determines the zinc content at the presynaptic terminals. Therefore, ZNT3 staining was performed to detect the abundance of LMTs in this study. Compared with the control group (Figure 3A), the labeling intensity of ZNT3 increased significantly in the subfield CA3 (Figures 3B–D) in both HT + 10D and HT + 14D groups. The relative positive area of ZNT3 in the in the subfield CA3 in each group was as follows: Control 1.03 (0.82, 1.16), HT + 10D 1.38 (1.29, 1.56), HT + 14D 1.29 (1.20, 1.42). Statistical analysis of ZNT3 also showed a significant increase of ZNT3 in subfields CA3 in both HT+ groups (Figure 3D) (**p1 = 0.001, *p2 = 0.022, *n* = 8 per group).

PSD95, a postsynaptic marker of glutamatergic synapses, is localized in the postsynaptic density, which keeps PSD95 from antibody penetrating and binding (26). Therefore, a modified pepsin protocol was performed to enhance the efficacy of staining. Compared with the control group, the abundance of PSD95 is not remarkably altered in subfields CA3 stratum lucidum (Figures 3E–G) in both HT + 10D and HT + 14D groups. The relative positive area of PSD95 in each group was as follows: Control 1.00 ± 0.16, HT + 10D 1.05 ± 0.22, HT + 14D 1.00 ± 0. Statistical analysis of PSD95 labeling density also showed no significant difference between HT+ and control groups (Figure 3H; *n* = 7 per group; *p*1 = 0.85, *p*2 = 0.99).

**Figure 4:**
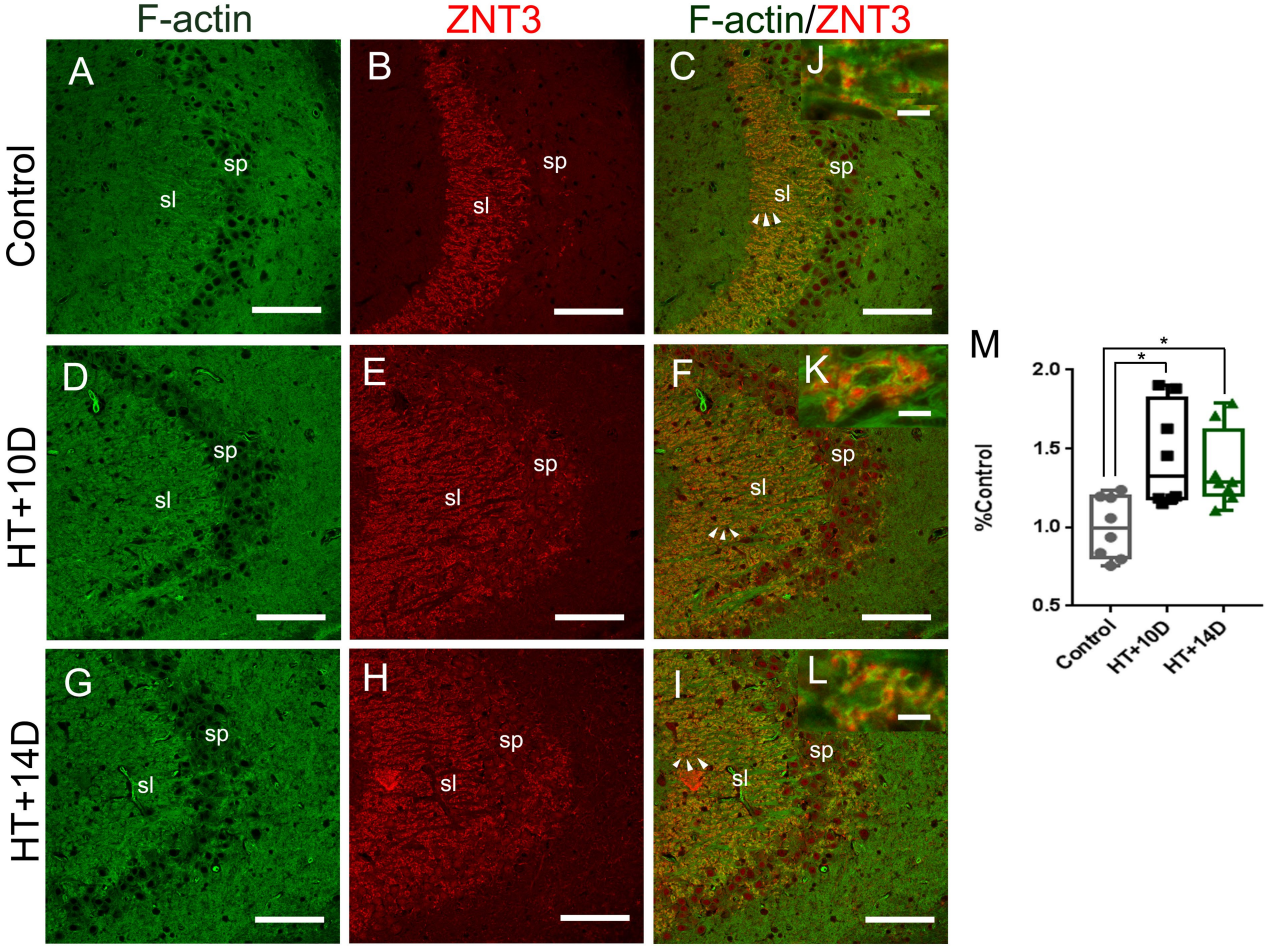
Relative localization of F-actin and ZNT3 in Hippocampal Subfield CA3. Scale bars: **(A–I)** 100 μm; **(J–L)** 5 μm. Representative images **(A,D,G)** (green) show F-actin puncta in the subfields of stratum lucidum of CA3 in different groups. Images **(B,E,H)** (red) show ZNT3 granules in the same region. Images **(C,F,I)** are merged from **(A,B,D,E,G,H)**, respectively. **(J–L)** Are magnified images for arrow areas. It shows that the localization of F-actin is roughly the same as that of ZNT3 in CA3 stratum lucidum. The fluorescence intensity of ZNT3 is dramatically increased **(E,H)** in the area where F-actin intensity is increased **(D,G)**. The enlarged images **(J–L)** show puncta of F-actin and ZNT3 are located in close proximity (yellow) indicating their co-localization. Statistical analysis of overlapping area of F-actin and ZNT3 (yellow) shows a significant increase in both HT+ groups **(M)** (*p1 = 0.03, *p2 = 0.02, *n* = 8 per group).

To examine whether there is correlation between the changes of F-actin cytoskeleton and LMTs, double labeling of phalloidin and ZNT3 was performed in different groups. It showed that the localization of F-actin was roughly the same as that of ZNT3 in CA3 stratum lucidum of hippocampus (Figures 4A-I). There was a dramatic increase of ZNT3 fluorescence intensity (Figures 4E,H) in the corresponding area where the intensity of F-actin was increased (Figures 4D,G) in HT + 10D and HT + 14D groups. Additionally, the enlarged images (Figures 4J–L) showed the puncta of ZNT3 were in close proximity to the puncta of F-actin (yellow), which indicated their co-localization. The relative overlapping area of F-actin and ZNT3 in each group was as follows: Control 1.00 (0.81, 1.19), HT + 10D 1.33 (1.18, 1.82), HT + 14D 1.27 (1.20, 1.61). Statistical analysis of overlapping area of F-actin and ZNT3 (yellow) showed a significant increase in both HT+ groups (Figures 4M) (*p1 = 0.03, *p2 = 0.02, *n* = 8 per group).

These results indicate that rearrangement of hippocampal F-actin cytoskeleton might mainly occur in the presynaptic terminals of mossy fiber synapses in the model of prolonged febrile seizures.

## 4. Discussion

Cumulative studies have shown that complex FSs cause a hyperexcitable state of the hippocampal network (2, 5, 27–29). The characteristics of this enhancement in hippocampal excitability after experimental FS are very similar to those of kindling epileptic models, both of which show that the animals do not have spontaneous seizures, but have a reduced seizure threshold to convulsants or electrical stimulations (5, 6). Remodeling of hippocampal F-actin cytoskeleton in PTZ chemical kindling epileptic model was proved in our previous study. F-actin significantly increased in the presynaptic terminals of MF-CA3 synapses, which led to the attenuation of gating effect of DG and the increase of excitatory output from DG to CA3 region (13). Accordingly, we focus on actin network again in this study to determine if the disturbance of the actin dynamics contribute to the hippocampal hyperexcitability after complex FSs.

### 4.1. Rearrangement of F-actin cytoskeleton in subfields of hippocampus after prolonged febrile seizures

Previous studies have reported the relationship between rearrangement of F-actin and epilepsy (12–14, 30, 31). Depolymerizing and stabilizing F-actin in rat hippocampus both led to epileptic seizures and long-term neuronal hyperexcitability (12). F-actin cytoskeleton was significantly reduced in area CA1 in the acute period of kainate model and pilocarpine model (30, 31). In addition, in the chronic epileptic model, F-actin showed differential rearrangement in the CA3 region, increased in presynaptic terminals in the PTZ induced chemical kindling model, and decreased dramatically in the postsynaptic spines in the pilocarpine induced post status epilepticus model (13, 14). Moreover, the application of FK506, dexamethasone, etc. in the critical period of epileptogenesis, was proved to protect the F-actin cytoskeleton, reduce the damage of synaptic structures, and contribute to decrease the spontaneous seizures in the chronic period of pilocarpine model (15, 16).

The results of this study showed that F-actin was up-regulated in the CA3 stratum lucidum of hippocampus after prolonged febrile seizures. The hippocampal subregion CA3 stratum lucidum is the main distribution site of MF-CA3 synapses. Therefore, we further analyzed the pre- and postsynaptic markers of MF-CA3 synapses. It showed that corresponding to the up-regulation of F-actin in this region, the abundance of presynaptic marker, ZNT3, increased significantly whereas the postsynaptic marker PSD95 did not change significantly. Further examination of overlapping area of F-actin and ZNT3 showed a significant increase in both HT+ groups. F-actin is the main cytoskeleton in the synapses, which is distributed both pre and post synaptic structures (11, 32, 33). Thus this result suggests that the change of F-actin might mainly occur in the presynaptic terminals of MF-CA3 synapses. F-actin is responsible for the organization, transportation and release of vesicles in presynaptic structures and participates in the initiation of presynaptic function (11). Changes of F-actin and ZNT3 in this study suggest an increase in presynaptic efficacy. MF-CA3 synapses are among one of the most powerful synapses in the brain (7). This synaptic remodeling in the present study is supposed to abate the filtering effect of dentate gyrus, increase the excitatory output and lead to enhanced susceptibility to epilepsy (34, 35).

### 4.2. The number of neurons in hippocampal subfields was unaltered after prolonged febrile seizures

Many studies suggest that the increase or decrease of the number of neurons in the hippocampus is closely related to the process of epileptogenesis. As previously reported, pilocarpine and kainate induced epileptic models both showed that neurons in CA3 and CA1 regions were obviously lost, while granular cells in dentate gyrus were obviously proliferating (14, 36–38). However, in the developing brain, there is less damage of hippocampal neurons than adult brain in kainate and pilocarpine treated post SE models (39, 40). In order to explore whether the change of F-actin in the FS model is caused by the increased or decreased number of neurons in this study, neurons in each subregion of the hippocampus were counted. The results showed that there was no significant increase or decrease in the number of neurons in each area of hippocampus after febrile seizures. This is consistent with previous reports that the hippocampal hyperexcitability in FS models does not depend on the alteration of the number of neurons (28). It also reflects the resistance of the developing brain to cell damage after convulsions as well. However, spontaneous seizures still occur in later life. This indicates that in the developing brain, the process of epileptogenesis may be more dependent on subtle structural changes such as synaptic circuit remodeling.

### 4.3. The rearrangement of F-actin cytoskeleton after prolonged febrile seizures is not age-dependent

Many studies suggest that febrile seizures occurring in younger children (less than 12 months or 18 months) have a higher risk of recurrence and further an increased risk of developing epilepsy (17–19). Therefore, we are curious whether this rearrangement of actin cytoskeleton after experimental febrile seizures is also age-dependent. In this study, the pups of 10 and 14 postnatal days were used, corresponding to the age of human being within and after one-year old (20). The results showed that there was no significant difference in rearrangement of actin cytoskeleton, cell count, and synaptic structure remodeling in hippocampal subregions between the two groups. The results of this study indicates that the rearrangement of F-actin cytoskeleton after febrile seizures in the developing brain may be more dependent on the recurrence and prolonged duration of seizures, but not on the age when febrile seizures occur.

## 5. Conclusion

Taken together, the rearrangement of F-actin cytoskeleton after prolonged FS was examined in the present study. It demonstrates that F-actin was significantly up-regulated in the stratum lucidum of hippocampal CA3 subregion and further analysis showed it corresponds the increase of the presynaptic marker of MF-CA3 synapses, which may enhance the excitatory output from the dentate gyrus to the CA3 region and contribute to the hippocampal hyperexcitability.

## Data availability statement

The datasets presented in this study can be found in online repositories. The names of the repository/repositories and accession number(s) can be found below: Baidu Netdisk, https://pan.baidu.com/s/18v87fgF79f8A-8g1CrNGWg, code: ksuf.

## Ethics statement

The animal study was reviewed and approved by the Research Ethics Committee of the First Hospital of Jilin University, Changchun, China (2020-0496).

## Author contributions

NY was responsible for immunohistochemistry experiments and confocal scanning. Y-BC was responsible for the supervision of the experimental process and the analysis of the results. Y-FZ was responsible for the experimental design and the manuscript writing. All authors contributed to the article and approved the submitted version.

## Funding

This study is funded by Department of Science and Technology of Jilin Province of China (20200201452JC, YDZ202201ZYTS090 and 20180101159JC).

## Conflict of interest

The authors declare that the research was conducted in the absence of any commercial or financial relationships that could be construed as a potential conflict of interest.

## Publisher’s note

All claims expressed in this article are solely those of the authors and do not necessarily represent those of their affiliated organizations, or those of the publisher, the editors and the reviewers. Any product that may be evaluated in this article, or claim that may be made by its manufacturer, is not guaranteed or endorsed by the publisher.
